# Hearing loss and healthcare expenditures in the United States: evidence of a public health market failure

**DOI:** 10.3389/fpubh.2026.1829845

**Published:** 2026-06-08

**Authors:** Yuval Arbel, Yifat Arbel, Netanel Kerner, Oriya Kerner

**Affiliations:** 1 Sir Harry Solomon School of Economics and Management, Western Galilee College, Akko, Israel; 2 Department of Mathematics, Bar Ilan University, Ramat Gan, Israel; 3 Faculty of Medicine, Hebrew University of Jerusalem, Jerusalem, Israel; 4 The Ruth and Bruce Rapoport Faculty of Medicine, Technion – Israel Institute of Technology, Haifa, Israel

**Keywords:** healthcare expenditures, hearing aid adoption, hearing loss, market failure, MEPS, public health economics

## Abstract

**Background:**

Hearing loss (HL) is one of the most prevalent chronic health conditions worldwide, affecting approximately 48 million adults in the United States. Despite the availability of effective treatment technologies, hearing aid adoption remains persistently low, with only 14–30% of individuals who could benefit from hearing aids actually using them. A growing body of evidence indicates that HL is associated with higher healthcare utilization, reduced labor market participation, cognitive decline, and diminished quality of life, implying substantial economic and societal costs.

**Objective:**

To integrate theoretical modeling with empirical analysis to estimate healthcare expenditure differentials associated with HL while accounting for the inability to distinguish treated from untreated HL in the MEPS dataset.

**Methods:**

Using the Medical Expenditure Panel Survey (MEPS) Public Use Files, we analyzed healthcare utilization and expenditures for individuals reporting HL. We develop a game-theoretic model in which hearing aid adoption is treated as a social dilemma, capturing private versus social costs. Because hearing aid utilization data were unavailable in this subset, the empirical analysis does not distinguish between treated and untreated HL. Regression models estimated associations between self-reported hearing difficulty and healthcare expenditures while controlling for demographic and socioeconomic characteristics.

**Results:**

HL was positively associated with higher healthcare utilization and expenditures across major service categories. Counterfactual predictions based on the weighted regression model suggest substantial expenditure differentials associated with HL. The absence of hearing aid utilization data implies that the analysis cannot directly estimate the potential expenditure reductions associated with treatment adoption.

**Conclusion:**

HL represents a substantial public health and economic burden. The theoretical model suggests that low hearing aid adoption may reflect a market failure arising from divergence between private and social incentives. The empirical findings quantify healthcare expenditure differentials associated with HL while acknowledging the limitation that treatment status is not observed. Future research incorporating hearing aid utilization data could provide more precise estimates of the economic implications of treatment adoption.

## 1 Introduction

Hearing loss is among the most prevalent chronic health conditions worldwide and a growing public health concern due to its association with cognitive decline, social isolation, higher healthcare utilization, and reduced quality of life (1). In the United States, approximately 48 million adults experience some degree of hearing impairment, with prevalence rising sharply with age. Globally, more than 430 million people require rehabilitation for disabling hearing loss (2). Despite the availability of effective treatment technologies, hearing aid adoption remains persistently low, with only 14–30% of individuals who could benefit actually using them (3).

Hearing loss is associated with a broad range of adverse outcomes, including dementia risk (4), depression and social isolation (5), and higher healthcare utilization (6). It also generates significant economic consequences, including reduced labor market participation and productivity losses (7). At the same time, hearing loss generates broader external costs through higher healthcare expenditures, reduced workforce productivity, and greater demand for long-term care services (2, 4, 7–9).

Despite these substantial costs, adoption remains limited. Individuals base decisions on private costs and perceived benefits, while social costs are not fully internalized (10–12). Behavioral barriers—such as stigma (13–15)—and information frictions further delay adoption.

Although prior research documents the health and economic burden of hearing loss (16), less attention has been given to the economic mechanisms sustaining this gap. This paper addresses this issue by developing a game-theoretic framework in which hearing aid adoption is modeled as a social dilemma, complemented by empirical analysis using MEPS data. Because hearing aid usage data were not available in this subset, our analysis does not differentiate between treated and untreated hearing loss, which limits interpretation regarding treatment effects.

This paper makes three main contributions to the literature. First, it develops a game-theoretic framework that conceptualizes hearing aid adoption as a social dilemma, highlighting the divergence between privately optimal decisions and socially efficient outcomes due to externalities. Second, it extends this framework using evolutionary dynamics to capture how adoption behavior evolves over time through social interactions, learning, and behavioral feedback. Third, it provides novel empirical evidence based on nationally representative MEPS data, estimating the association between hearing loss and healthcare expenditures and estimating healthcare expenditure differentials associated with hearing loss. By integrating theoretical modeling with empirical analysis, the paper offers a unified perspective on the economic mechanisms underlying the persistent gap between clinical need and treatment uptake. Accordingly, the theoretical framework should be interpreted as exploratory and assumption-dependent rather than as a direct empirical test of treatment effects.

The remainder of the paper is organized as follows. Section 2 reviews the literature on hearing loss, hearing aid adoption, and the associated economic consequences. Section 3 presents the game-theoretic framework and analyzes the equilibrium and evolutionary dynamics of hearing aid adoption. Section 4 presents the econometric analysis using MEPS data and estimates the incremental healthcare expenditures associated with hearing impairment. Section 5 concludes with a discussion of the policy implications of the findings and outlines directions for future research.

## 2 Literature review and conceptual background

Hearing loss has been extensively studied across a range of disciplines, including epidemiology, public health, audiology, and health economics. The existing literature consistently shows that hearing impairment is not only a clinical condition but also a broader social and economic challenge with important implications for healthcare systems and labor markets. Previous research has examined the prevalence of hearing loss, patterns of hearing aid adoption, the economic consequences of untreated hearing impairment, and the policy mechanisms that may promote treatment uptake.

A large body of epidemiological research documents both the high prevalence of hearing loss and the persistent gap between clinical need and actual treatment adoption. According to the Centers for Disease Control and Prevention (CDC), nearly 48 million adults in the United States are estimated to have measurable hearing impairment (17). Despite the availability of effective hearing technologies, adoption rates remain relatively low, with only about 14–30% of individuals who could benefit from hearing aids actually using them (18). Adoption rates are generally higher at older ages, particularly among individuals aged 65 and older, yet even within this group usage remains substantially below clinical need. Evidence from Europe and East Asia indicates that similar adoption gaps are observed across different healthcare systems, consistent with low hearing aid uptake being a widespread global phenomenon rather than a country-specific issue (19). Overall, these findings highlight the persistence of a substantial gap between treatment availability and actual utilization.

Beyond prevalence, an expanding literature has examined the broader economic consequences associated with untreated hearing loss. Empirical evidence indicates that hearing impairment is associated with higher healthcare utilization and medical expenditures. Untreated hearing loss has been linked to a greater incidence of hospitalizations, falls, and complications related to chronic conditions, generating estimated direct medical costs ranging from $3 to $13 billion annually in the United States (20). More recent studies further indicate that untreated hearing loss is associated with higher overall healthcare spending, while hearing aid use is associated with lower healthcare costs over time (e.g., (8, 9)). In addition to direct healthcare costs, hearing impairment generates substantial productivity losses in the labor market. Individuals with untreated hearing loss are more likely to experience reduced labor force participation, lower workplace productivity, and earlier retirement, with annual productivity losses estimated at approximately $30–35 billion in the United States (7). Moreover, the association between hearing impairment and cognitive decline is consistent with the possibility of untreated hearing loss may contribute to higher long-term care costs related to dementia and other neurocognitive conditions (4). Taken together, this body of evidence indicates that hearing loss imposes significant economic burdens that extend well beyond direct treatment costs.

Another important strand of the literature focuses on the behavioral and market factors that contribute to the low adoption of hearing aids. One key barrier is the high upfront cost of hearing devices, which can discourage adoption, particularly among individuals with limited financial resources. In many markets, hearing aids cost between approximately $1,500 and $6,000 per device (18). In addition to financial barriers, information asymmetry plays a central role in limiting treatment uptake. Many individuals underestimate the potential benefits of hearing aids or delay seeking treatment because they view hearing loss as a normal part of aging rather than a treatable medical condition (5). Delays in help-seeking behavior are also common, with individuals often postponing treatment for several years after the onset of symptoms (21). Expectations regarding device effectiveness and prior experiences with hearing care services further influence adoption decisions (22). Social stigma may further discourage adoption, as some individuals perceive hearing aids as a visible sign of aging or disability (14). Recent research highlights that stigma can operate through both self-perception and social interactions, reinforcing avoidance behavior and reducing willingness to adopt hearing technologies (15, 23). These findings indicate that hearing aid adoption is associated not only with economic costs but also with behavioral and social considerations.

Given the substantial social costs associated with untreated hearing loss, a number of studies have examined policy interventions aimed at increasing treatment adoption. One approach involves financial mechanisms such as subsidies or expanded insurance coverage for hearing aids. Evidence indicates that such policies are associated with higher adoption rates, particularly among older adults (16). Another approach focuses on behavioral interventions designed to reduce informational and psychological barriers to treatment. Insights from behavioral economics indicate that policies such as default screening programs and automatic referrals can increase participation in preventive health services by lowering decision costs and reducing procrastination (12). More recently, regulatory reforms have aimed to improve access to hearing technologies, including the introduction of over-the-counter (OTC) hearing aids, which have the potential to reduce costs and increase market competition (24).

Despite the breadth of this literature, existing research has largely examined the clinical, behavioral, and economic dimensions of hearing loss in isolation. Relatively few studies have attempted to integrate these perspectives within a unified analytical framework that explicitly models hearing aid adoption decisions while accounting for the external social costs of untreated hearing loss. In particular, the strategic interaction between individual decision-making and broader social welfare implications has received limited formal attention.

The present study contributes to this literature by developing a game-theoretic framework that models hearing aid adoption as a social dilemma in which individual incentives may diverge from socially optimal outcomes due to externalities and behavioral frictions. By combining theoretical modeling with empirical estimation of healthcare expenditure associations, this paper provides a more comprehensive analysis of the economic mechanisms underlying the persistent gap in hearing aid adoption.

While the existing literature clearly documents the health and economic consequences of hearing loss, relatively little work has developed formal economic models that explain why the gap between treatment availability and actual adoption persists. In particular, the interaction between private decision-making and the broader social costs associated with untreated hearing loss remains insufficiently explored. Understanding this interaction is essential for evaluating whether observed adoption patterns reflect individually rational behavior or a broader market failure. To address this issue, the next section develops a game-theoretic framework in which hearing aid adoption is modeled as a strategic decision problem that incorporates both private incentives and external social costs.

## 3 Theoretical framework: game-theoretic model of hearing aid adoption

### 3.1 Pure strategy dynamics

We model hearing aid adoption as a strategic interaction in which the privately optimal decision of the individual may differ from the socially optimal outcome. The consumer chooses between two pure strategies: adopting a hearing aid (A) or not adopting (N). Although this decision is made on the basis of private costs and benefits, it also has broader implications for society through its effect on future healthcare utilization and public expenditures. The model therefore captures a social dilemma: an individual may rationally choose not to adopt a hearing aid even when adoption would generate positive net benefits for society as a whole.

#### 3.1.1 Payoff structure

Let B denote the private benefit from improved hearing and functioning and let C denote the private cost of obtaining and using a hearing aid. In addition, let S represent the social benefit associated with lower long-term healthcare expenditures when hearing loss is treated, and let E denote the external cost imposed on society when hearing loss remains untreated.

If the individual adopts a hearing aid, the private payoff is B−C , while society obtains a payoff of S . If the individual does not adopt, the private payoff is normalized to zero, but society incurs a payoff of −E , reflecting the additional burden associated with untreated hearing loss. The resulting payoff structure is presented in Table 1.

**Table 1:** Social dilemma in hearing aid adoption: payoff matrix.

Consumer decision	Consumer payoff	Social payoff
Adopt (A)	B − C	S
Not Adopt (N)	0	−E

This payoff matrix illustrates the core tension of the model. The consumer evaluates adoption only in terms of the private net return B−C , whereas society also accounts for the external effects of untreated hearing loss. As a result, the individually rational choice may fail to internalize the broader social costs, giving rise to a divergence between private incentives and collective welfare (10–12).

#### 3.1.2 Nash equilibrium

The individual adopts a hearing aid if and only if the private payoff from adoption is positive, that is, when B−C>0 . If instead B−C<0 , the individual chooses not to adopt, since non-adoption yields a higher private payoff than adoption.

From a social perspective, however, adoption is optimal whenever the total welfare effect is positive, namely when

B−C+S>0

The social dilemma arises in the case where


B−C<0<B−C+S

Under this condition, the Nash equilibrium is non-adoption, because the individual does not take the social benefit S into account when making the decision. Yet this equilibrium is socially sub-optimal, since adoption would increase total welfare once the social gains from lower long-term healthcare costs are considered. In other words, the equilibrium outcome is stable from the standpoint of individual incentives, but inefficient from the standpoint of society. This gap between private rationality and social efficiency provides the theoretical basis for policy intervention, such as subsidies or public provision, to encourage hearing aid adoption.

### 3.2 Evolutionary game model (extension of the pure-strategy model)

The evolutionary game model presented here extends the pure-strategy framework described in Section 3.1. While the pure-strategy model assumes that individuals make fixed, one-time decisions, the evolutionary model accounts for dynamic adaptation and social interactions over time, allowing us to capture the feedback effects and strategy evolution within populations.

In this framework, individuals repeatedly interact within a social environment, and their strategies evolve according to replicator dynamics, whereby more successful strategies proliferate while less successful ones decline. This approach is particularly relevant to hearing loss behaviors, as decisions regarding hearing aid adoption, disclosure of hearing impairment, and social engagement are influenced by both personal outcomes and societal interactions.

We visualize the model dynamics using phase diagrams, which summarize the evolution of strategy frequencies over time. The phase diagrams (Table 2) highlight the stable and unstable equilibria, showing how social pressures, stigma, and interaction patterns affect the likelihood of adopting proactive behaviors, such as using hearing aids or engaging in supportive social networks.

**Table 2:** Equilibrium points and strategy transitions in the evolutionary game model.

Variable	Interpretation
*x* = 0	Unstable: If the population starts at zero adoption, it tends to move away from this point.
*x* = 0.3	Stable interior equilibrium: Trajectories starting below 0.3 increase, and above 0.3 decrease, converging to this value.
*x* = 1	Unstable: If the population reaches full adoption, small deviations cause it to move away from full adoption.

**Figure 1:**
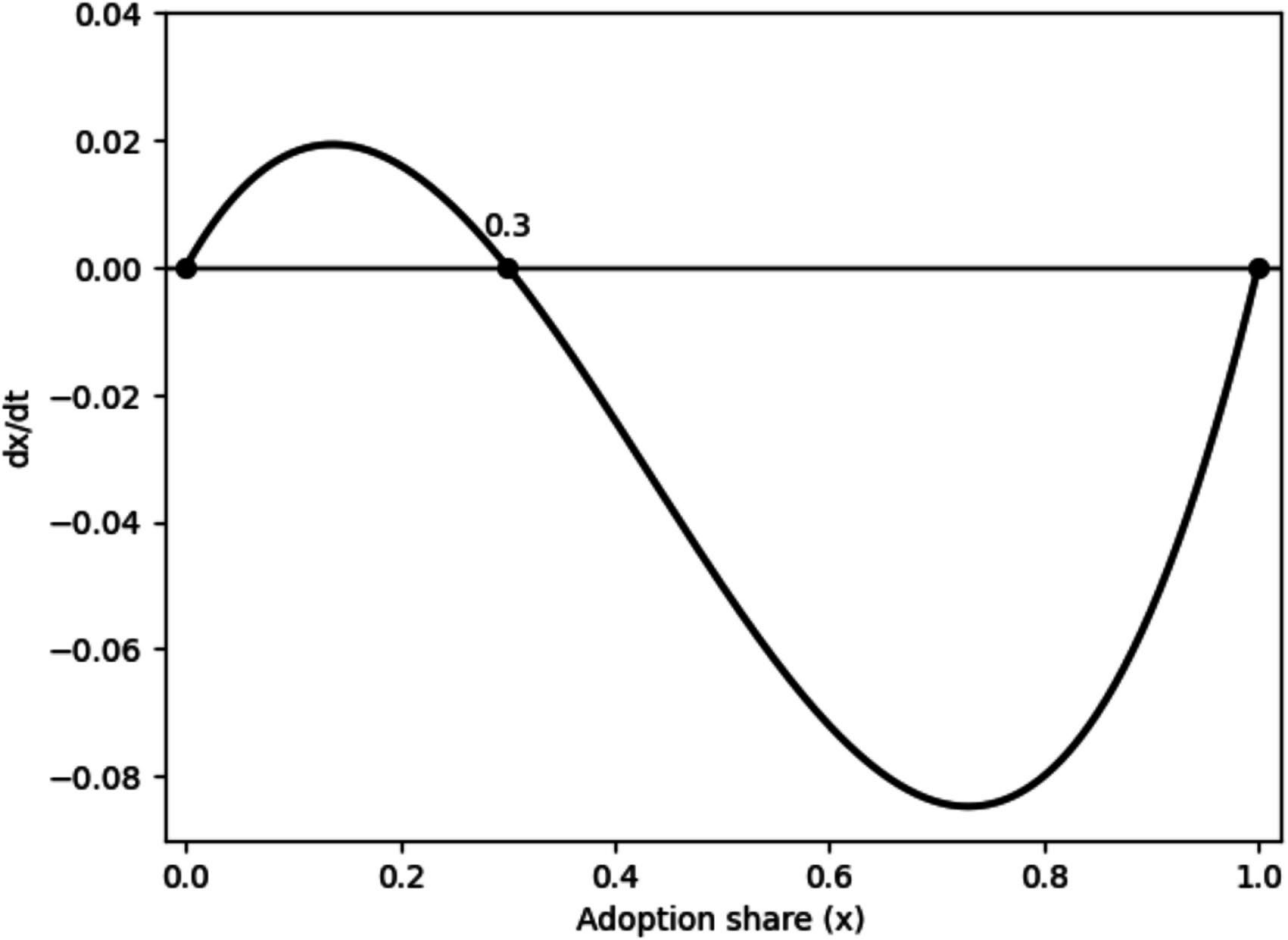
Phase diagram of evolutionary adoption dynamics.

For clarity, the mathematical derivations underpinning the replicator dynamics are presented in Supplementary Appendices A1, A2, while the main text focuses on interpreting the behavioral outcomes and policy-relevant insights derived from the phase diagrams (see Figure 1).

In the evolutionary game model, the variable x represents the fraction of the population that adopts hearing aids. The dynamics, $\dot{x}$ , indicate how this fraction changes over time depending on the strategies’ relative success.

The table below presents the original numerical values derived from the phase diagram in the evolutionary game model. These values indicate the key equilibrium points and thresholds that determine how strategy frequencies evolve over time.

#### 3.2.1 Intuitive meaning

- At x=0 : Very few people adopt hearing aids. Here, $\dot{x}>0$ , meaning that the adoption fraction tends to increase. If the population starts at zero, social influences and the payoff structure push adoption upward toward the interior equilibrium ( $x^{*}\approx 0.3$ ), rather than staying at zero. Thus, x=0 is unstable because the population naturally moves away from it.
- At x=1 : Everyone adopts hearing aids. Here, $\dot{x}<0$ , meaning the system moves away from full adoption, again due to relative payoffs and social effects. Full adoption is also unstable.
- At the interior equilibrium $x^{*}$ : For $x<x^{*}$ , $\dot{x}>0$ (adoption grows), and for $x>x^{*}$ , $\dot{x}<0$ (adoption shrinks). This creates a “restoring force” that stabilizes the population at $x^{*}$ . Intuitively, if only 20% of the population uses hearing aids, the dynamics push adoption toward 30%. If 40% use them, adoption tends to decrease toward 30%. Hence, $x^{*}$ is stable.

#### 3.2.2 Summary

The phase diagram visually shows how the population moves from extreme adoption states toward the stable interior equilibrium. This captures both the social interaction effects and the impact of stigma: adoption increases when too few use aids and decreases when too many do, eventually stabilizing at a socially balanced adoption rate.

### 3.3 Social welfare and the optimal adoption rate

**Figure 2:**
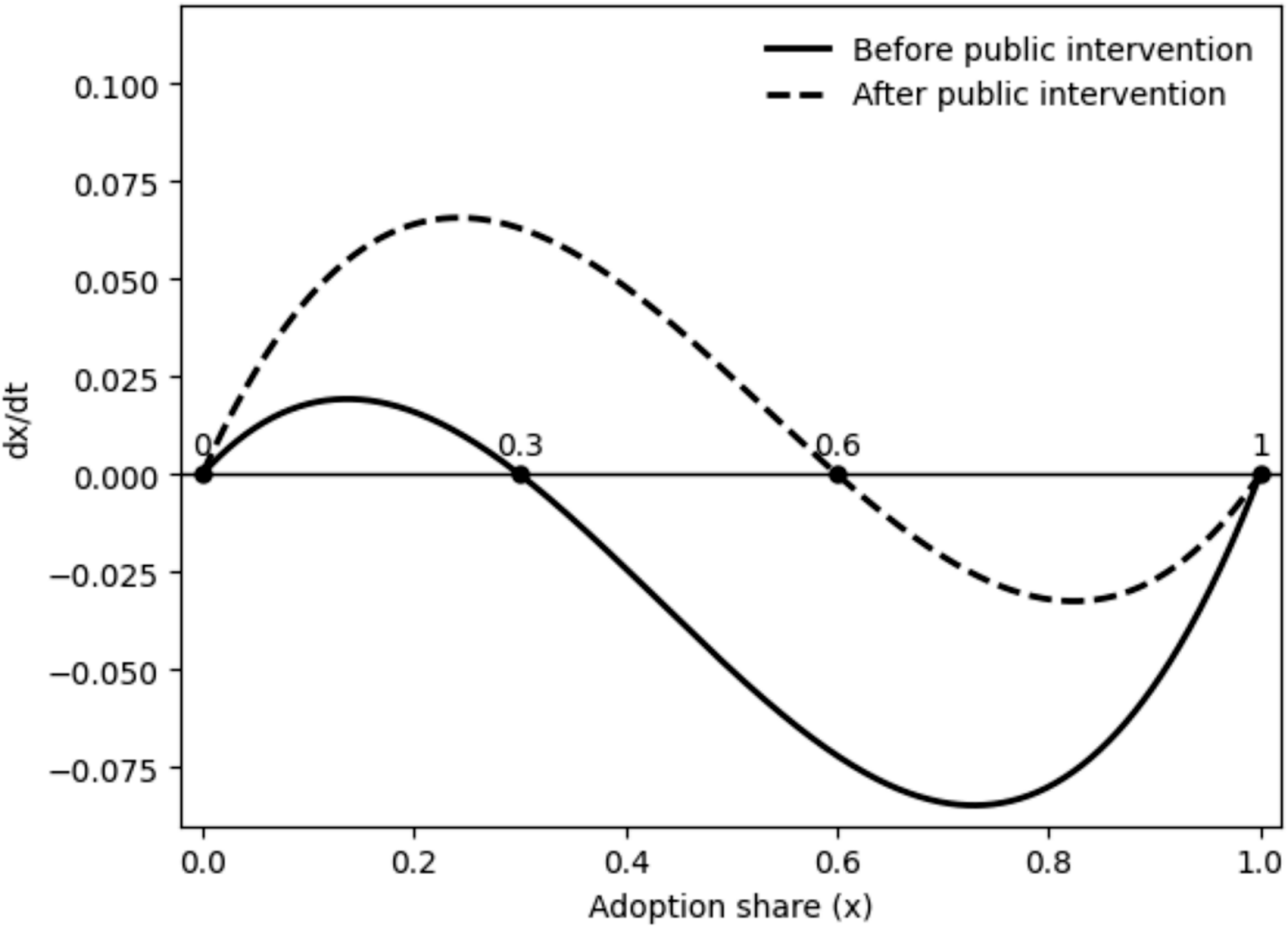
Policy-induced shift in the evolutionary adoption dynamics.

Suppose that $x^{*}=\frac{B-C+\alpha }{\beta }=0.3$ and $x^{\mathrm{opt}}=\frac{B-C+S+E}{\delta }=0.6$ (for a mathematical derivation see Supplementary Appendices B, C). The objective of the policy planner will be to shift the phase diagram so that the stable equilibrium will be achieved at $x^{\mathrm{opt}}=0.6$ . This point is exemplified in Figure 2.

### 3.4 Public policy influence on adoption dynamics

The phase diagram (Figure 2) illustrates how the proportion of individuals adopting proactive hearing behaviors evolves over time. In the absence of policy interventions, the system tends toward an equilibrium at x ≈ 0.3, meaning about 30% of the population adopts these behaviors under current social and economic conditions.

Public policy acts as a catalyst that enhances incentives, reduces barriers, or provides support for hearing aid adoption. Intuitively, this can be seen as “lifting” the payoff of adopting proactive strategies. In the phase diagram, this influence shifts the original black curve upward to the dashed curve, representing a higher attractiveness of adoption at all adoption levels. As a result, the new equilibrium moves to x ≈ 0.6, effectively doubling the adoption rate.

This upward shift is achieved by policies that:

1. Subsidize hearing devices or reduce costs.
2. Higher public awareness, reducing stigma.
3. Encourage social support networks that reinforce adoption.

A simplified table (Table 3) can summarize the dynamics.

**Table 3:** Effect of public policy on hearing aid adoption rates.

Curve	Equilibrium adoption rate	Interpretation
Black (no policy)	0.3	Baseline adoption
Dashed (with policy)	0.6	Optimal adoption rate under public policy

This explanation connects the formal phase diagram to intuitive policy effects: the dashed curve shows that policy changes make adoption more rewarding, pulling the population toward a higher adoption level.

## 4 Statistical and econometric analysis

### 4.1 Data source and empirical design

The empirical analysis uses data from the Medical Expenditure Panel Survey (MEPS), a nationally representative survey of the civilian noninstitutionalized population in the United States conducted by the Agency for Healthcare Research and Quality (AHRQ). MEPS collects detailed information on healthcare utilization, expenditures, insurance coverage, health status, and socioeconomic characteristics (25, 26).

This study uses individual-level data from the 2023 Full-Year Consolidated Data File and therefore adopts a cross-sectional empirical specification rather than exploiting the longitudinal structure of MEPS. Healthcare expenditures are measured using the variable TOTEXP, representing total annual medical expenditures across all payment sources, including out-of-pocket payments, private insurance, Medicare, and Medicaid. Hearing impairment is identified using the MEPS variable DFHEAR, which indicates whether an individual reports serious difficulty hearing. Additional controls include age, family income, and education. Because hearing aid usage data were unavailable in this subset, the empirical analysis does not distinguish between treated and untreated hearing loss.

The analytic sample is restricted to adults aged 18 years and older with non-missing information on healthcare expenditures, hearing difficulty, income, education, and survey-design variables. After applying these restrictions, the final analytic sample includes approximately 6,490 individuals.

To account for the complex sampling structure of MEPS, all descriptive statistics and regression models were estimated using survey-weighted procedures. Specifically, the analysis applies the person-level sampling weight (LONGWT), primary sampling unit (VARPSU), and sampling strata (VARSTR). Survey weighting adjusts for unequal probabilities of selection, clustering, and stratification, thereby ensuring that the estimates are representative of the U.S. population (25, 26). This procedure adjusts estimates and standard errors for unequal probabilities of selection, stratification, and clustering. The final analytic sample includes 6,490 adults, representing a weighted population size of approximately 257.25 million individuals.

### 4.2 Descriptive statistics

Table 4 reports the descriptive statistics of variables that are later incorporated into the empirical model.

**Table 4:** Variable definitions and weighted descriptive statistics.

Panel A: Variable definitions	
Variable	Definition
Healthcare	Annual individual healthcare expenditures (USD).
Hearing loss	Indicator variable equal to 1 if the individual reports serious difficulty hearing, and 0 otherwise.
Age	Age of the individual in years.
Income	Annual family income in U.S. dollars.

Panel B: Survey-weighted descriptive statistics				
Variable	Observations	Weighted Mean	Std. Error	95% Confidence interval
Healthcare	6,490	8,330.41	313.98	7,710.08–8,950.73
Hearing loss	6,490	0.045	0.003	0.039–0.051
Age	6,490	48.26	0.40	47.47–49.05
Income	6,490	106,801	2445.40	101,969.70–111,632.40
Education	6,490	13.58	0.07	13.45–13.72

Statistics are survey-weighted using MEPS sampling weights, strata, and PSUs. The weighted prevalence of hearing loss in the analytic sample is approximately 4.5%.

Panel A of Table 4 presents the definitions of the variables used in the analysis. Healthcare expenditures represent annual individual medical spending measured in U.S. dollars. Hearing loss is defined as a binary indicator equal to one if the respondent reports serious difficulty hearing and zero otherwise. Age is measured in years, while income represents annual family income in U.S. dollars. These definitions clarify the construction of the key variables used in both the descriptive statistics and the regression analysis.

Panel B of Table 4 presents survey-weighted descriptive statistics for the primary variables included in the analysis using a sample of 6,490 adults from the Medical Expenditure Panel Survey (MEPS). Mean annual healthcare expenditures were estimated at $8,330 (SE = 313.98), with a 95% confidence interval ranging from $7,710 to $8,951. Approximately 4.5% of respondents reported serious hearing difficulty, based on the weighted prevalence estimate.

The average age of participants was 48.3 years (SE = 0.40), while mean annual family income was approximately $106,801 (SE = 2,445.40), with a 95% confidence interval between $101,970 and $111,632. Respondents also reported an average of 13.6 years of education.

Overall, the descriptive statistics indicate substantial variation in healthcare expenditures and income across individuals. All estimates were calculated using MEPS sampling weights, strata, and primary sampling units (PSUs) to ensure national representativeness.

### 4.3 Empirical model and results

#### 4.3.1 Empirical model

The empirical analysis examines the association between hearing loss and healthcare expenditures using survey-weighted regression analysis. The baseline specification is given by:


$$\begin{array}{l} \ln(\text{Healthcar}e_{i}+1)=\alpha +\beta_{1}\text{HearingLos}s_{i}+\beta_{2}\mathrm{Ag}e_{i}+\\ \beta_{3}\ln(\text{Incom}e_{i}+1)+\\ \beta_{4}\text{Educatio}n_{i}+\varepsilon_{i} \end{array}$$

where $\text{Healthcar}e_{i}$ represents annual healthcare expenditures for individual i , measured in U.S. dollars; $\text{HearingLos}s_{i}$ is an indicator variable equal to 1 if the respondent reports serious difficulty hearing and 0 otherwise; $\mathrm{Ag}e_{i}$ denotes age in years; $\text{Incom}e_{i}$ represents annual family income; and $\text{Educatio}n_{i}$ measures years of completed schooling. The term α denotes the intercept, $\beta_{1}-\beta_{4}$ are the parameters to be estimated, and $\varepsilon_{i}$ captures unobserved determinants of healthcare expenditures.

The inclusion of demographic and socioeconomic controls helps account for observable differences between individuals with and without hearing loss that may influence healthcare expenditures. Because MEPS is based on a complex survey design, all descriptive statistics and regression estimates were calculated using survey weights, primary sampling units, and sampling strata to obtain nationally representative estimates and appropriate standard errors.

Healthcare expenditures and income are highly right-skewed, with a relatively small number of respondents reporting very large values. To reduce skewness, stabilize the variance, and facilitate proportional interpretation of coefficients, the model applies logarithmic transformations to both healthcare expenditures and income. Specifically, the dependent variable is defined as $\ln(\text{Healthcar}e_{i}+1)$ , while income enters the model as $\ln(\text{Incom}e_{i}+1)$ .

Adding one before taking logarithms allows observations with zero expenditures or zero income to remain in the estimation sample. This is particularly relevant in survey-based healthcare data, where zero values are relatively common. Retaining these observations improves representativeness and avoids excluding economically meaningful segments of the population. Similar approaches are commonly used in analyses of MEPS data and healthcare expenditures.

#### 4.3.2 Results

Table 5 reports the survey-weighted and unweighted OLS regression estimates for the determinants of healthcare expenditures, where the dependent variable is the natural logarithm of annual healthcare expenditures plus one. The weighted model explains approximately 17.4% of the variation in healthcare expenditures (R^2^ = 0.1736), while the unweighted robust model explains 19.7% (R^2^ = 0.1965). Although the explanatory power is moderate, the statistically significant F-statistics indicate that the included covariates jointly contribute meaningfully to explaining healthcare expenditures. As noted by Kmenta (27), regression models based on cross-sectional household data commonly yield R^2^ values around 0.20 because of substantial individual heterogeneity that cannot be fully captured by observable characteristics.

**Table 5:** Survey-weighted and unweighted regression results for log healthcare expenditures.

Variables	Survey-weighted model	Unweighted robust model
	(1) weighted_main ln_healthcare	(2) unweighted_robust ln_healthcare
Hearing loss	0.824***	0.743***
	(1.47 × 10^−6^)	(6.37 × 10^−10^)
Age	0.0640***	0.0676***
	(<0.01)	(<0.01)
ln_income	0.0632*	0.0371*
	(0.0774)	(0.0645)
Education	0.205***	0.192***
	(<0.01)	(<0.01)
Constant	−0.0185	0.360
	(0.967)	(0.186)
Observations	6,490	6,490
*R*-squared	0.1736	0.1965
*F*-statistic	207.17	417.52
DF (numerator, denominator)	(4,149)	(4, 6,485)

Dependent variable: Log annual healthcare expenditures.

Survey-weighted estimates are based on MEPS sampling weights, strata, and PSUs. Robust standard errors are reported in parentheses. Coefficients are reported with *p*-values in parentheses. The weighted specification uses the complex survey design implemented with MEPS probability weights. Statistical significance: ***p < 0.01, **p < 0.05, *p < 0.10.

Hearing loss is strongly and positively associated with healthcare expenditures. In the survey-weighted specification, the coefficient on hearing loss is 0.824 (*p* < 0.01), implying that individuals reporting serious hearing difficulty incur substantially higher healthcare expenditures than those without hearing loss. Applying the exact semi-log transformation $e^{0.824}-1$ , indicates that individuals with hearing loss experience approximately 128% higher healthcare expenditures, holding other covariates constant. Age, income, and education are also positively associated with healthcare expenditures, suggesting that older, wealthier, and more educated individuals tend to spend more on healthcare services. Because the analysis is cross-sectional, the estimated coefficients should be interpreted as associations rather than causal effects.

#### 4.3.3 Robustness checks

To verify the reliability of the main findings, we compared the survey-weighted and unweighted regression estimates reported in Table 5. In the survey-weighted specification, the coefficient on hearing loss is 0.824 (p < 0.01), implying that individuals reporting hearing loss incur approximately 128% higher healthcare expenditures $(e^{0.824}-1)\times 100$ . In the unweighted robust model, the coefficient declines slightly to 0.743 (p < 0.01), corresponding to approximately 110% higher healthcare expenditures $(e^{0.743}-1)\times 100$ . Thus, although the magnitude of the association is somewhat smaller in the unweighted specification, hearing loss remains strongly and positively associated with healthcare expenditures across both models. The consistency in statistical significance and effect size suggests that the main findings are robust to alternative estimation approaches and heteroskedasticity adjustments.

Additionally, Q-Q plots and residual histograms used to assess approximate normality are presented in Supplementary Appendix D, confirming the validity of standard inference procedures. Together, these robustness checks indicate that the observed association between hearing loss and healthcare expenditures is reliable and not sensitive to alternative specifications.

#### 4.3.4 Incremental healthcare expenditures associated with hearing loss

To evaluate the economic burden associated with hearing loss, we estimate the incremental healthcare expenditures implied by the regression model using a counterfactual prediction framework. Specifically, for each individual in the sample, predicted healthcare expenditures are computed under two scenarios: (i) the observed case in which the individual reports hearing loss, and (ii) a counterfactual scenario in which hearing loss is set to zero while all other observed characteristics remain unchanged. The difference between these predicted outcomes represents the individual-level estimated expenditure differential associated with hearing loss.

Because hearing aid utilization data were unavailable, the estimates reflect average associations with self-reported hearing difficulty and do not distinguish between treated and untreated hearing loss.

The results from the survey-weighted regression model reported in Table 5 indicate that hearing loss is positively and significantly associated with higher healthcare expenditures. The weighted specification incorporates MEPS sampling weights, strata, and primary sampling units and is therefore representative of the approximately 257.25 million individuals in the noninstitutionalized United States population represented during the study period. Controlling for age, income, and education, individuals reporting hearing loss exhibit substantially higher predicted healthcare expenditures relative to otherwise comparable individuals without hearing loss.

Using the weighted prediction procedure described in Supplementary Appendix E, the estimated incremental healthcare expenditure associated with hearing loss is approximately $30,321 per person annually. The distribution of predicted expenditure differentials exhibits substantial heterogeneity across individuals, reflecting demographic and socioeconomic variation within the sample. Nevertheless, the positive average differential suggests that hearing loss is associated with meaningfully greater healthcare utilization and spending.

Supplementary Appendix E provides a detailed description of the estimation procedure, including the logarithmic specification of the regression model, retransformation of predicted values into dollar terms, construction of the counterfactual predictions, and derivation of the aggregate national expenditure differential.

To illustrate the potential macroeconomic magnitude of this association, the weighted prevalence of serious hearing difficulty in the sample—approximately 4.5%—was applied to the representative U.S. population of roughly 257.25 million individuals. This extrapolation implies that approximately 11.6 million Americans may experience serious hearing difficulties. Multiplying this population estimate by the predicted incremental annual expenditure yields an illustrative aggregate healthcare expenditure differential of approximately $351.0 billion annually.

This estimate should be interpreted cautiously. It reflects overall differences in healthcare expenditures associated with hearing loss rather than direct treatment costs attributable exclusively to hearing impairment. Consequently, the estimates likely capture broader patterns of healthcare utilization associated with comorbidities, functional limitations, and other health conditions correlated with hearing loss rather than narrowly defined hearing-related medical expenditures alone.

## 5 Conclusion, policy implications, and limitations

This study examines the persistent gap in hearing aid adoption by combining a theoretical (game-theoretic and evolutionary) framework with empirical evidence. The regression results show that individuals with hearing loss incur significantly higher healthcare expenditures, underscoring the healthcare expenditure differentials associated with hearing loss. While these results do not identify causal mechanisms, they are consistent with the theoretical model. The empirical findings indicate that hearing loss is associated with substantially higher healthcare expenditures, suggesting that theoretical benefits of treatment may be substantial. However, despite these benefits, hearing aid adoption remains low. This gap between high potential gains and limited adoption is consistent with a framework in which individual decisions are shaped not only by financial costs but also by informational frictions and behavioral factors, which may prevent individuals from fully internalizing the benefits of adoption and the broader social costs of non-adoption.

The regression results indicate the reported R^2^ (0.197) is relatively low, reflecting the heterogeneity in healthcare expenditures and unobserved factors. For context, Kmenta (27) notes that a typical R^2^ for various household functions from the University of Michigan’s Survey Research Center data is close to 0.20, indicating that much of the variation across households is due to factors beyond the explanatory variables. Despite the modest R^2^, the estimated associations are statistically significant and substantively meaningful. The natural logarithm of the dependent variable allows interpretation of coefficients as percentage changes in healthcare expenditures associated with hearing loss and covariates, while stabilizing variance and reducing skewness. Moreover, the model’s *F*-value (207.17, df₁ = 4, df₂ = 149) is highly significant, confirming the joint explanatory power of the four variables (Hearing loss, Age, Log income, Education).

These points reinforce that, although substantial unobserved variation remains, hearing loss is robustly associated with higher healthcare expenditures. Additional robustness checks using heteroskedasticity-robust standard errors confirm that the associations are not sensitive to unequal residual variance. Visual inspection of residuals via Q-Q plots or histograms further supports approximate normality, ensuring that standard inference remains valid. Together, these diagnostics indicate that the reported effect sizes are reliable and meaningful for both individual-level interpretation and extrapolation to population-level economic burden.

The evolutionary extension of the theoretical model further suggests that individual decisions are interdependent: when adoption is low, social interactions may reinforce non-use, contributing to the persistence of stigma and low uptake. This provides a theoretical basis for understanding how non-financial barriers can emerge and persist alongside financial constraints.

The study highlights the substantial economic and public health burden associated with hearing loss in the United States. Individuals reporting serious hearing difficulty incur approximately 110% higher healthcare expenditures, corresponding to an incremental cost of $30,321 per person annually. Extrapolated to the estimated 11.6 million affected adults, this implies a population-level burden of roughly $351.0 billion per year. However, these estimates are based on an empirical model that does not differentiate between individuals who use hearing aids and those who do not, so the potential mitigating role of treatment in healthcare costs is not accounted for. This limitation underscores the need for cautious interpretation regarding the societal significance of under-adoption of hearing aids.

From a policy perspective, the relative importance of barriers to hearing aid adoption is likely to vary across healthcare systems. In more market-based systems, such as the United States, out-of-pocket costs, limited insurance coverage, and price transparency are likely to play a larger role. In contrast, in countries with stronger public financing or broader coverage of hearing care, financial barriers may be less binding, while non-financial factors—such as stigma, delayed diagnosis, limited awareness, and weaker referral pathways—may account for a larger share of the adoption gap. In this sense, the present findings may have broader cross-country relevance: although the institutional sources of under-adoption differ, both financial and behavioral barriers can sustain inefficiently low uptake. Accordingly, policies should be tailored to healthcare-system design and should combine financial support with interventions aimed at information, screening, and stigma reduction.

### 5.1 Limitations

Several limitations should be acknowledged when interpreting the findings. First, although the analysis employs appropriate MEPS survey-weighting procedures, including probability weights, strata, and primary sampling units (PSUs), the study remains observational and cross-sectional in nature. Consequently, the estimates should be interpreted as nationally representative associations rather than causal effects. Because exposures and outcomes are measured contemporaneously, the analysis cannot establish temporal ordering between hearing loss and healthcare expenditures, and residual confounding from unobserved health conditions or behavioral factors may remain. Future research using longitudinal survey designs would allow researchers to better examine the dynamic relationship between hearing impairment, healthcare utilization, and hearing aid adoption over time, while improving causal inference and reducing concerns regarding reverse causality.

Second, hearing impairment is measured using self-reported serious hearing difficulty rather than objective audiometric testing. The dataset also does not contain information regarding the severity, duration, or progression of hearing impairment, factors that may affect healthcare utilization patterns and expenditure levels. Future research using longitudinal and clinically validated hearing measures could provide more precise estimates of the relationship between hearing impairment and medical spending.

Third, the empirical analysis does not directly observe behavioral mechanisms emphasized in the theoretical framework, including stigma, informational frictions, delayed treatment-seeking behavior, and social perceptions regarding hearing aid adoption. Consequently, these mechanisms should be interpreted as conceptual pathways rather than directly estimated empirical effects.

Finally, the current MEPS subset does not include information on hearing aid ownership or utilization. As a result, the empirical estimates capture average healthcare expenditure differentials associated with self-reported hearing difficulty in the population and cannot isolate the potential moderating effect of treatment adoption.

## Data Availability

The data used in this study are publicly available from the Medical Expenditure Panel Survey (MEPS), administered by the Agency for Healthcare Research and Quality (AHRQ). The datasets can be accessed at: https://meps.ahrq.gov/mepsweb/.
