# Supplementary material for: Hearing loss and healthcare expenditures in the United States: evidence of a public health market failure
Source: Front Public Health. 2026 Jun 8;14:1829845. doi: 10.3389/fpubh.2026.1829845 (PMC13283996; doi:10.3389/fpubh.2026.1829845)
Supplement: Supplementary file 1 [file Data_sheet_1.pdf]

Supplementary Material

Appendix A1: Evolutionary Mixed-Strategy Dynamics

The Nash equilibrium analysis presented above identifies the static incentive structure governing hearing-aid adoption. However, real-world behavioral adjustment rarely occurs instantaneously. Individuals gradually adapt their decisions through social learning, imitation, and observation of relative payoffs. To capture this dynamic adjustment process, we extend the model using an evolutionary game framework in which the share of adopters evolves over time.

Let $x \in [0,1]$ denote the proportion of individuals in the population who adopt hearing aids, while $1-x$ represents the proportion who do not adopt. In this interpretation, x may also be viewed as the probability of adoption in a mixed-strategy population. The payoff from adoption is assumed to depend on the prevalence of adoption in the population, reflecting social interactions, learning effects, and behavioral feedback.

Specifically, the expected private payoff from adoption is defined as

$$\pi_A(x) = B - C + \alpha - \beta x,$$

while the payoff from non-adoption is

$$\pi_N = 0.$$

Here, B represents the private benefit from improved hearing, C denotes the private cost of acquiring and maintaining hearing aids, $\alpha > 0$ captures baseline behavioral or informational incentives encouraging adoption, and $\beta > 0$ represents diminishing marginal incentives as adoption becomes widespread. The term $\alpha - \beta x$ therefore introduces frequency dependence into the payoff structure.

The parameter α represents baseline incentives that encourage adoption, while $\beta > 0$ captures diminishing marginal incentives as adoption becomes more widespread. In practice, individuals who adopt early are typically those who derive the largest private benefits from treatment, such as individuals with more severe hearing loss or those whose professional or social activities rely heavily on effective communication. As adoption increases, the remaining non-adopters are more likely to consist of individuals with milder symptoms, lower perceived benefits, or stronger behavioral barriers such as stigma or denial. Consequently, the marginal incentive to adopt declines with the share of adopters in the population, which is reflected by the term $-\beta x$. This assumption generates diminishing marginal incentives and leads to the existence of an interior equilibrium adoption rate.

Let the average payoff in the population be

$$\bar{\pi}(x) = x\pi_A(x) + (1 - x)\pi_N.$$

Following standard evolutionary game theory, the adjustment of the adoption rate is described by the replicator dynamic

$$\dot{x} = x(\pi_A(x) - \bar{\pi}(x)).$$

Because $\pi_N = 0$, the dynamic simplifies to

$$\dot{x} = x(1 - x)\pi_A(x),$$

which yields

$$\dot{x} = x(1 - x)(B - C + \alpha - \beta x).$$

This equation governs the evolution of hearing-aid adoption in the population. Strategies that generate above-average payoffs become more prevalent over time, while strategies yielding below-average payoffs decline.

Appendix A2: Evolutionary Phase Diagram

The evolutionary dynamics of hearing-aid adoption can be illustrated using a phase diagram defined on the unit interval $x \in [0,1]$, where $x$denotes the proportion of individuals who adopt hearing aids. Recalling that the replicator dynamic governing the adjustment process is

$$\dot{x} = x(1-x)(B - C + \alpha - \beta x).$$

Stationary points occur when

$$\dot{x} = 0,$$

which yields three equilibria:

$$x_1 = 0, x_2 = 1, x_3 = x^* = \frac{B - C + \alpha}{\beta}.$$

An interior equilibrium exists whenever

$$0 < \frac{B - C + \alpha}{\beta} < 1.$$

To determine stability, consider the sign of $\dot{x}$. When $x < x^*$, the term $B - C + \alpha - \beta x$is positive, implying $\dot{x} > 0$and an increasing share of adopters. When $x > x^*$, the term becomes negative, implying $\dot{x} < 0$and a declining adoption rate. Consequently, trajectories on both sides move toward the interior equilibrium.

Figure 1 illustrates the evolutionary phase diagram of the adoption dynamic $\dot{x} = x(1 - x)(a - bx)$. The horizontal axis represents the share of adopters x, while the vertical axis represents the growth rate of adoption $\dot{x}$. The points where the curve intersects the horizontal axis correspond to stationary states $x = 0$, $x = x^*$, and $x = 1$. We calibrated the figure so that $x^* = \frac{B-C+\alpha}{\beta} = 0.3$ (see Appendix B). For values of $x < x^*$, the dynamic is positive ($\dot{x} > 0$), implying that the share of adopters increases over time. For $x > x^*$, the dynamic becomes negative ($\dot{x} < 0$), causing the adoption rate to decline. Consequently, the interior equilibrium $x^*$is stable, while the boundary equilibria $x = 0$ and $x = 1$are unstable.

Figure 1: Phase Diagram of Evolutionary Adoption Dynamics

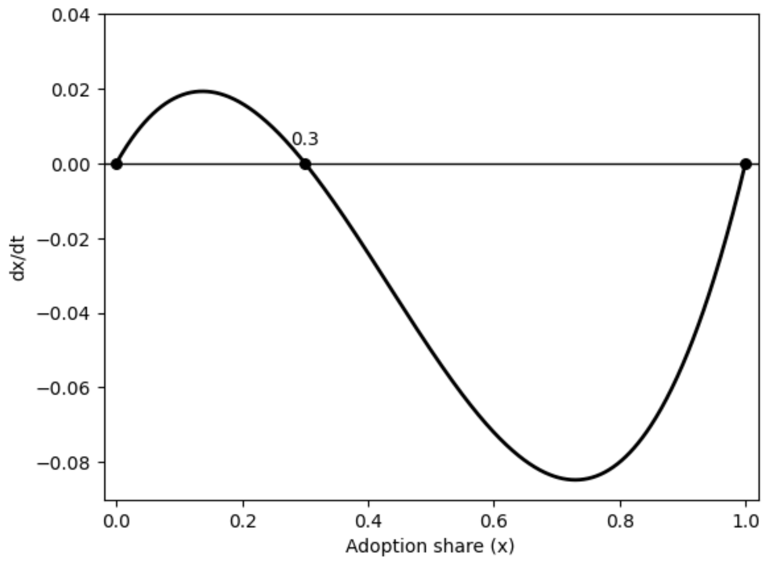

Stability is determined by the sign of $\dot{x}$on either side of each stationary point. Near $x = 0, \dot{x} > 0$, so trajectories move away from zero, implying that $x = 0$is unstable. Near $x = 1$, $\dot{x} < 0$, so trajectories move away from one, implying that $x = 1$is also unstable. By contrast, at the interior equilibrium x^*, $\dot{x} > 0$for $x < x^*$and $\dot{x} < 0$for $x > x^*$, so trajectories converge to x^*, which is therefore stable.

Appendix B: Social Welfare and the Optimal Adoption Rate

While the evolutionary equilibrium reflects private incentives, the socially optimal adoption rate must account for externalities associated with untreated hearing loss. Let the social welfare function be

$$W(x) = x(B - C + S) - (1 - x)E - \frac{\delta}{2}x^2,$$

where S represents the social benefit from adoption (such as reduced healthcare costs and improved productivity), Edenotes the external cost imposed by untreated hearing loss, and $\delta > 0$ captures diminishing marginal social returns from additional adoption.

The socially optimal adoption rate maximizes welfare:

$$\max_x W(x).$$

The first-order condition is

$$\frac{dW}{dx} = B - C + S + E - \delta x = 0,$$

which yields the welfare-maximizing adoption rate

$$x^{opt} = \frac{B - C + S + E}{\delta}.$$

Because

$$\frac{d^2W}{dx^2} = -\delta < 0,$$

this solution represents a maximum.

Suppose that $x^* = \frac{B-C+\alpha}{\beta} = 0.3$ and $x^{opt} = \frac{B-C+S+E}{\delta} = 0.6$ (see this calibration in Appendix A). The objective of the policy planner will be to shift the phase diagram so

that the stable equilibrium will be achieved at $x^{opt} = 0.6$. This point is exemplified in Figure 2:

Figure 2: Policy-Induced Shift in the Evolutionary Adoption Dynamics

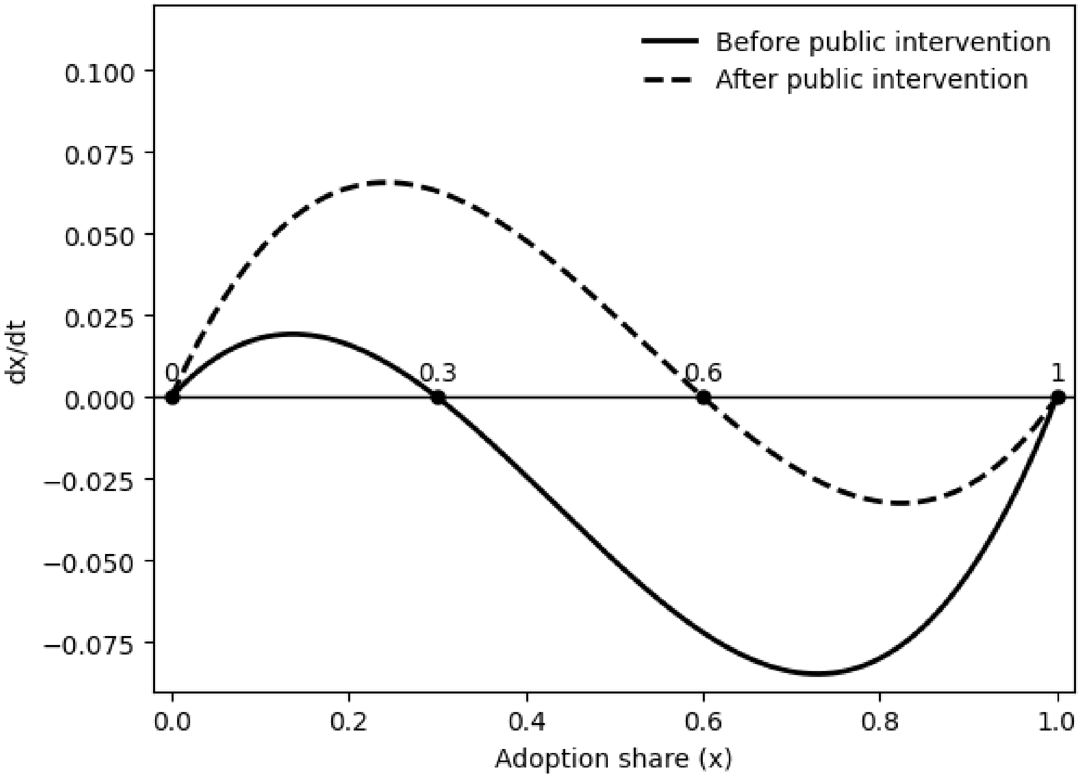

Appendix C. Illustrative Calibration of the Adoption Equilibrium

Without loss of generality

$$x^* = \frac{B - C + \alpha}{\beta} = 0.3 \text{ and } x^{opt} = \frac{B - C + S + E}{\delta} = 0.6.$$

One convenient numerical choice is:

$$B - C = 0.1, \alpha = 0.2, \beta = 1,$$

so that

$$x^* = \frac{0.1 + 0.2}{1} = 0.3.$$

Then choose

$$S + E = 0.5, \delta = 1,$$

which gives

$$x^{opt} = \frac{0.1 + 0.5}{1} = 0.6.$$

To model policy intervention, keep $B - C = 0.1$and $\beta = 1$, but increase $\alpha$from $0.2$to 0.5. Then the post-intervention evolutionary equilibrium becomes

$$x^*_{\text{after}} = \frac{0.1 + 0.5}{1} = 0.6.$$

So, the interpretation is:

- **before intervention**: decentralized equilibrium $x^* = 0.3$
- **Social optimum**: $x^{opt} = 0.6$
- **after intervention**: policy shifts private incentives, so the new stable equilibrium moves to 0.6

Appendix D: Q-Q Plot of Regression Residuals

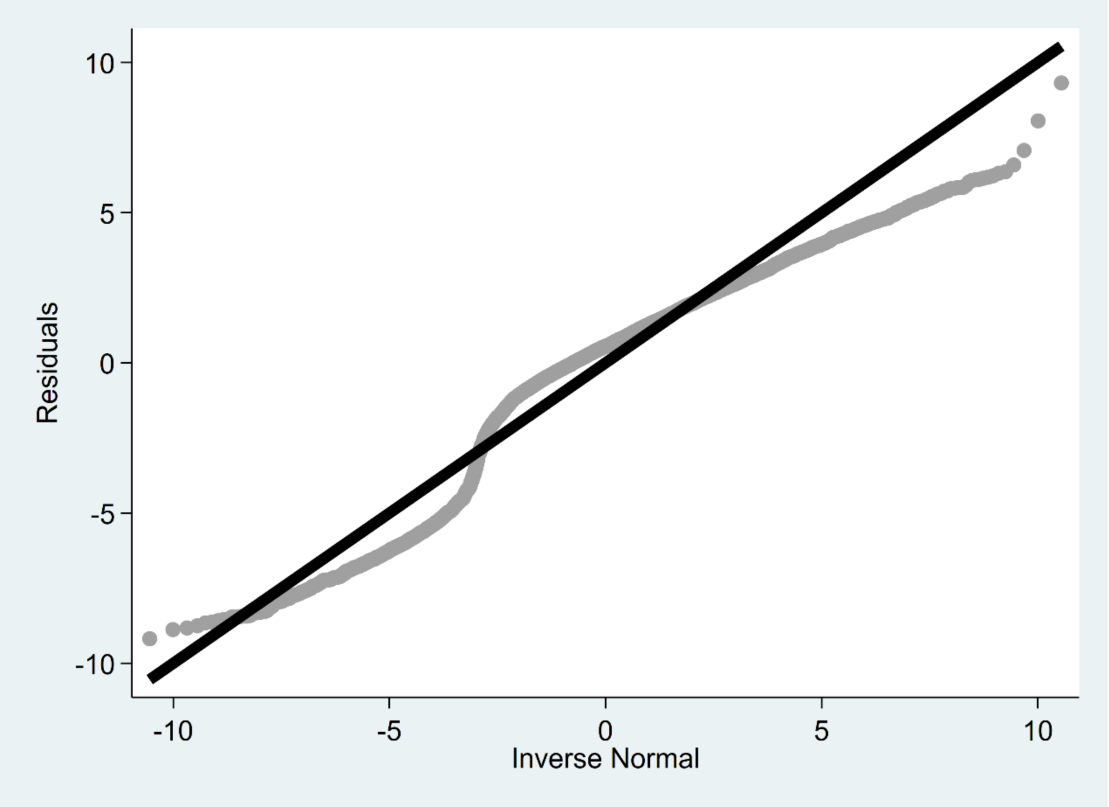

Note: The Q-Q plot in this appendix displays the residuals from the regression analysis of healthcare expenditures. It is used to assess whether the residuals approximately follow a normal distribution, which is an underlying assumption of the regression model. While the plot suggests approximate normality, minor deviations are expected due to the heterogeneity in household healthcare expenditures.

Appendix E. Derivation of Incremental Healthcare Expenditures Associated with Hearing Loss

This appendix describes the procedure used to derive the estimated incremental healthcare expenditures associated with hearing loss. Because healthcare expenditures are highly right-skewed, the main analysis estimates a log-linear regression model of annual healthcare expenditures, following standard approaches used in healthcare expenditure analysis.

The estimated survey-weighted regression model is:

$$\ln(\text{Healthcare}_i + 1) = \beta_0 + \beta_1 \text{HearingLoss}_i + \beta_2 \text{Age}_i + \beta_3 \ln(\text{Income}_i + 1) + \beta_4 \text{Education}_i + \varepsilon_i$$

The preferred specification is the survey-weighted model reported in Table 5 because it incorporates MEPS sampling weights, strata, and primary sampling units (PSUs). Accordingly, the estimates are representative of approximately 257.25 million noninstitutionalized U.S. residents during the study period.

For each individual, predicted healthcare expenditures were calculated under two scenarios:

$$\hat{Y}_i^{HL=1} = \exp(\widehat{\ln Y_i} \mid HearingLoss_i = 1)$$

$$\hat{Y}_i^{HL=0} = \exp(\widehat{\ln Y_i} \mid HearingLoss_i = 0)$$

All other covariates—age, income, and education—were held at their observed values. The individual-level incremental expenditure was then calculated as:

$$\Delta_i = \hat{Y}_i^{HL=1} - \hat{Y}_i^{HL=0}$$

The weighted mean of these individual differences produced an estimated incremental healthcare expenditure of approximately \$30,321 per person annually.

The weighted prevalence of serious hearing difficulty in the analytic sample was approximately 4.5%. Applying this prevalence estimate to the represented U.S. population of 257.25 million individuals implies that approximately 11.6 million individuals report serious hearing difficulty (257,248,697×0.045=11,576,191).

The aggregate annual healthcare expenditure differential was therefore calculated as:

$$11{,}576{,}191 \times \$30{,}321 \approx 351{,}033{,}000{,}000 \approx 351 \text{ billion annually}$$

This estimate should be interpreted cautiously. It reflects overall healthcare expenditure differentials associated with hearing loss rather than direct medical costs attributable exclusively to hearing impairment. The estimate likely captures broader healthcare utilization associated with comorbidities, functional limitations, and correlated health conditions rather than narrowly defined hearing-related treatment costs alone.
