# Supplementary material for: Hearing loss and healthcare expenditures in the United States: evidence of a public health market failure
Source: Front Public Health. 2026 Jun 8;14:1829845. doi: 10.3389/fpubh.2026.1829845 (PMC13283996; doi:10.3389/fpubh.2026.1829845)
Supplement: Supplementary file 2 [file Data_sheet_2.pdf]

```
                                                                                 ___  ____  ____  ____  ____(R)
                                                                                /__    /   ____/   /   ____/
                                                                               ___/   /   /___/   /   /___/
                                                                                 Statistics/Data analysis

                                                                                   User: Dr. yuval Arbel
          Project: Does Vertical Urban Development Improve or Worsen Public Health? Evidence from
 1 . gen healthcare = TOTEXPY2
         name:  <unnamed>
          log:  C:\Users\yuvalr\Documents\Lamas_datasets_via_huji\Lung Cancer - Melanoma - Breast Cancer - Yuval - M
     log type:  smcl
    opened on:  10 May 2026, 19:28:02

 2 . do "C:\Users\yuvalr\Documents\Lamas_datasets_via_huji\Lung Cancer - Melanoma - Breast Cancer - Yuval - Miri - Y

 3 . set more off

 4 .
 5 . cd "C:\Users\yuvalr\Documents\Lamas_datasets_via_huji\Lung Cancer - Melanoma - Breast Cancer - Yuval - Miri - Y
   C:\Users\yuvalr\Documents\Lamas_datasets_via_huji\Lung Cancer - Melanoma - Breast Cancer - Yuval - Miri - Yifat\M

 6 .
 7 . import excel "Book1.xlsx", sheet("Sheet1") firstrow clear
   (12 vars, 8,292 obs)

 8 .
 9 . * Main variables
10 . gen healthcare = TOTEXPY2

11 . replace healthcare = . if TOTEXPY2 < 0
   (117 real changes made, 117 to missing)

12 .
13 . gen hearingloss = DFHEAR4

14 . replace hearingloss = 0 if DFHEAR4 == 2
   (7,640 real changes made)

15 . replace hearingloss = . if DFHEAR4 < 0
   (250 real changes made, 250 to missing)

16 .
17 . gen AGE = AGEY2X

18 . replace AGE = . if AGEY2X == -1 | AGEY2X < 18
   (1,735 real changes made, 1,735 to missing)

19 .
20 . gen Income = FAMINCY2

21 . replace Income = . if FAMINCY2 < 0
   (118 real changes made, 118 to missing)

22 . gen ln_income = ln(Income + 1)
   (118 missing values generated)

23 .
24 . gen education = EDUCYR
```

```
25 . replace education = . if EDUCYR < 0
   (544 real changes made, 544 to missing)

26 .
27 . gen ln_healthcare = ln(healthcare + 1)
   (117 missing values generated)

28 .
29 . * Keep adult analytic sample with valid survey design variables
30 . keep if AGE >= 18
   (0 observations deleted)

31 . keep if LONGWT > 0 & VARPSU < . & VARSTR < .
   (0 observations deleted)

32 .
33 . * Declare MEPS complex survey design
34 . svyset VARPSU [pweight=LONGWT], strata(VARSTR) singleunit(centered)

         pweight: LONGWT
             VCE: linearized
     Single unit: centered
        Strata 1: VARSTR
            SU 1: VARPSU
           FPC 1: <zero>

35 .
36 . * Survey-weighted descriptive statistics
37 . svy: mean healthcare hearingloss AGE Income education
   (running mean on estimation sample)

   Survey: Mean estimation

   Number of strata =     105      Number of obs   =        6,490
   Number of PSUs   =     257      Population size =  257,248,697
                                   Design df       =          152
```

	Mean	Linearized Std. Err.	[95% Conf.	Interval]
healthcare	**8330.406**	**313.978**	**7710.082**	**8950.731**
hearingloss	**.0450374**	**.0031948**	**.0387255**	**.0513493**
AGE	**48.26121**	**.3981589**	**47.47457**	**49.04785**
Income	**106801**	**2445.399**	**101969.7**	**111632.4**
education	**13.58271**	**.0673229**	**13.4497**	**13.71571**

```
38 . svy: proportion hearingloss
   (running proportion on estimation sample)

   Survey: Proportion estimation

   Number of strata =     105      Number of obs   =        8,042
   Number of PSUs   =     257      Population size =  327,554,118
                                   Design df       =          152
```

	Proportion	Linearized Std. Err.	Logit [95% Conf.	Interval]
hearingloss				
0	**.9630631**	**.0026001**	**.9575683**	**.9678703**
1	**.0369369**	**.0026001**	**.0321297**	**.0424317**

```
.
. * Main survey-weighted regression
. svy: regress ln_healthcare hearingloss AGE ln_income education
```

(running **regress** on estimation sample)

Survey: Linear regression

Number of strata	= **105**	Number of obs	=	**6,490**
Number of PSUs	= **257**	Population size	=	**257,248,697**
		Design df	=	**152**
		F(**4**, **149**)	=	**207.17**
		Prob > F	=	**0.0000**
		R-squared	=	**0.1736**

ln_healthc~e	Coef.	Linearized Std. Err.	t	P>|t|	[95% Conf.	Interval]
hearingloss	**.8244315**	**.1644421**	**5.01**	**0.000**	**.4995442**	**1.149319**
AGE	**.064029**	**.0028086**	**22.80**	**0.000**	**.05848**	**.0695779**
ln_income	**.0631739**	**.0355305**	**1.78**	**0.077**	**-.0070236**	**.1333714**
education	**.2047259**	**.0198502**	**10.31**	**0.000**	**.1655081**	**.2439438**
_cons	**-.0184706**	**.4431669**	**-0.04**	**0.967**	**-.8940329**	**.8570916**

```
.
. * Store estimates
. estimates store weighted_main

.
. * Optional: unweighted comparison
. reg ln_healthcare hearingloss AGE ln_income education, vce(robust)
```

Linear regression

Number of obs	=	**6,490**
F(4, 6485)	=	**417.52**
Prob > F	=	**0.0000**
R-squared	=	**0.1965**
Root MSE	=	**2.9232**

ln_healthc~e	Coef.	Robust Std. Err.	t	P>|t|	[95% Conf.	Interval]
hearingloss	**.7431562**	**.1200508**	**6.19**	**0.000**	**.5078169**	**.9784954**
AGE	**.0675818**	**.0020096**	**33.63**	**0.000**	**.0636422**	**.0715213**
ln_income	**.0370905**	**.0200606**	**1.85**	**0.065**	**-.0022348**	**.0764159**
education	**.1921361**	**.0131606**	**14.60**	**0.000**	**.1663371**	**.2179352**
_cons	**.359729**	**.2719752**	**1.32**	**0.186**	**-.1734322**	**.8928901**

```
48 . estimates store unweighted_robust

49 .
50 . * Export results
51 . outreg2 [weighted_main unweighted_robust] using Table1_weighted.xls, replace pval
   Table1_weighted.xls
   dir : seeout

52 .
   end of do-file

53 . log close
         name:  <unnamed>
          log:  C:\Users\yuvalr\Documents\Lamas_datasets_via_huji\Lung Cancer - Melanoma - Breast Cancer - Yuval - M
     log type:  smcl
    closed on:  10 May 2026, 19:28:13
```
